# Supplementary material for: Multi-context genetic modeling of transcriptional regulation resolves novel disease loci
Source: Nat Commun. 2022 Sep 28;13:5704. doi: 10.1038/s41467-022-33212-0 (PMC9519579; doi:10.1038/s41467-022-33212-0)
Supplement: Supplementary file 1 — Supplementary Information [file 41467_2022_33212_MOESM1_ESM.pdf]

Supplementary Information

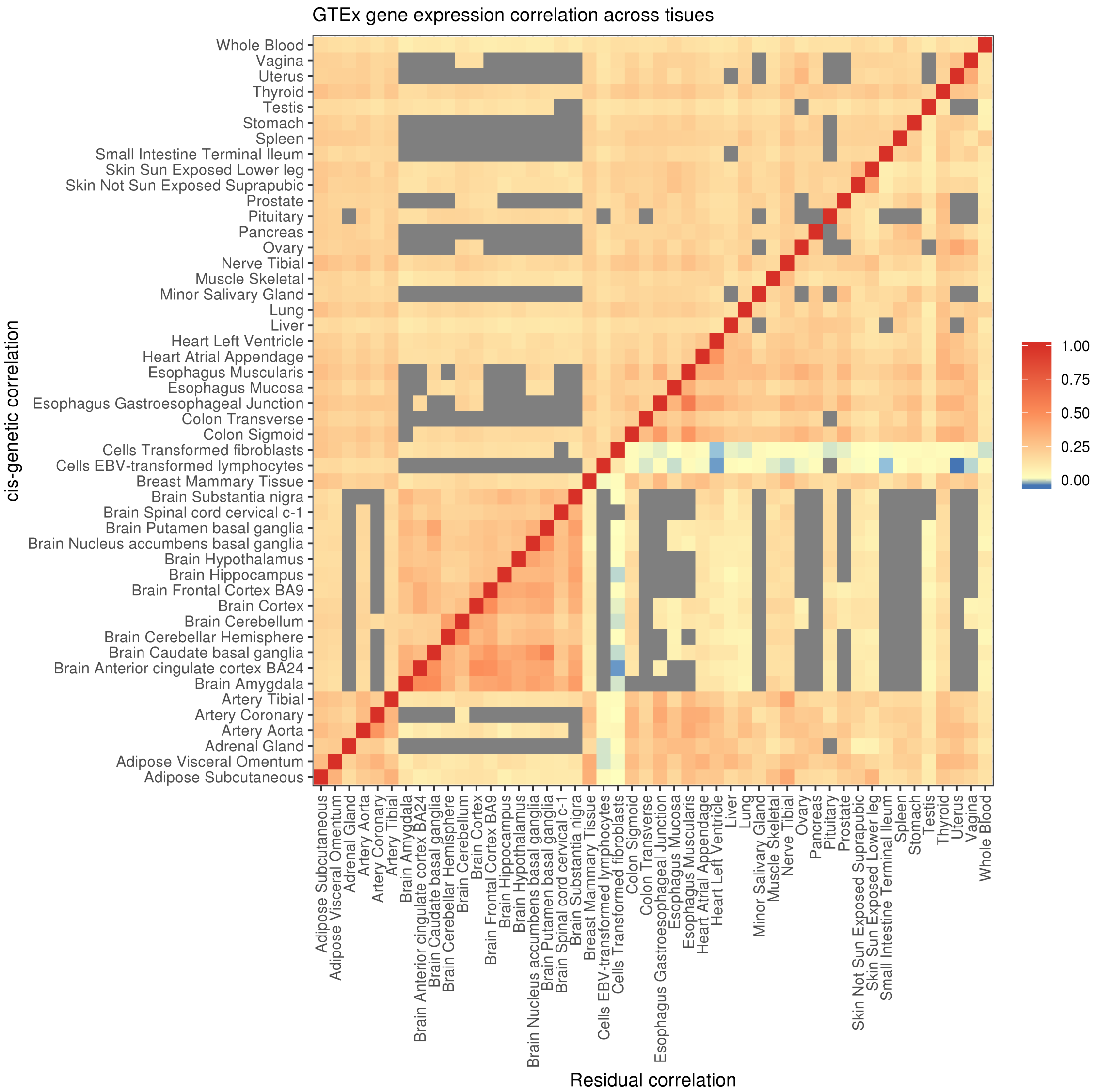

Supplementary Figure 1. Gene expression correlation across tissues in the GTEx study. Using a linear mixed model with bivariate REML [1, 2], we calculated cis-genetic and residual (which captures variance due to both trans-genetic effects as well as residual effects) variance and covariance components for each gene-tissue pair across GTEx. The gray units indicate tissue pairs with less than 10% sample overlap. In both the genetic (upper) and residual (lower) components, there was widespread cis-genetic and residual correlation, with the brain tissues showing higher correlations compared to other tissues.

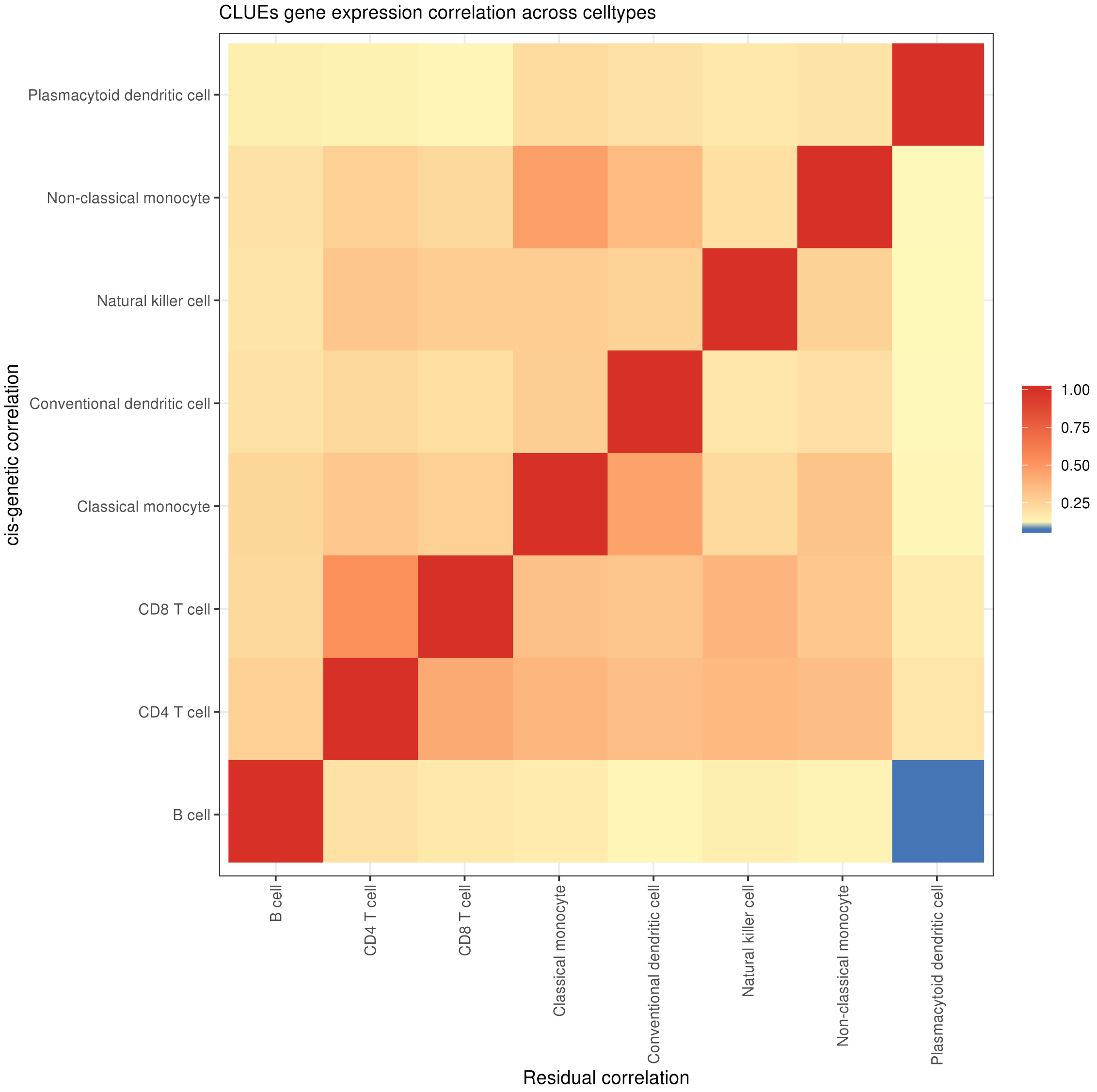

Supplementary Figure 2. Gene expression correlation across cell types in the CLUEs study. Using a linear mixed model with bivariate REML[1, 2], we calculated cis-genetic and residual (which includes trans-genetic effects) variance and covariance components for each gene-cell type pair across CLUEs.

Supplementary Methods

Intuition for using the decomposition to model genomic features The decomposition described in the methods section lays a framework for CONTENT as it directly accounts for the shared noise and generates orthogonal context-shared and context-specific components of genomic features. First, we note that in multi-context data, repeated measurements of one individual will likely have correlated errors; in the context of GTEx data, an individual's environment as well as technical noise is likely to affect their expression in all contexts. The above decomposition exploits this structure, which improves the power to learn the context-specific variability of expression. Put more rigorously, consider the expression of gene j in an individual measured in a baseline context and then again after a stimulation:

$$E_{ij1} = \mathbf{g_i}\beta_j + \epsilon_{ij1} \tag{1}$$

$$E_{ij2} = \mathbf{g_i}\beta_j + \mathbf{g_i}\gamma_j + \epsilon_{ij2} \tag{2}$$

Where E_{ij1} and E_{ij2} denote the observed expression level of individual i at gene j at baseline and stimulation respectively, $\mathbf{g_i}$ represents a vector of the individuals' genotype at some nearby cis-SNPs, β_j denotes the baseline genetic effects on expression, γ_j denotes the stimulation-related genetic effects on expression, and ϵ_{ij1} and ϵ_{ij2} represent the environmental effects (or noise) on the individual's expression of gene j in baseline and stimulation respectively. In teasing apart the genetic effects that are different after stimulation, one might examine the difference in the expression between contexts:

$$E_{ij2} - E_{ij1} = \mathbf{g_i}\beta_j + \mathbf{g_i}\gamma_j + \epsilon_{ij2} - \mathbf{g_i}\beta_j - \epsilon_{ij1} \tag{3}$$

$$= \mathbf{g_i}\gamma_j + \epsilon_{ij2} - \epsilon_{ij1} \tag{4}$$

which leaves only the difference in expression due to the stimulation-specific, or in other words, context-specific component, and noise. Under the scenario in which the errors are perfectly correlated, (4) simplifies to:

$$E_{ij2} - E_{ij1} = \mathbf{g_i}\gamma_j \tag{5}$$

Clearly, this will greatly increase our ability to build a genetic model of the stimulation-specific component. In terms of CONTENT, the baseline genetic effects correspond to the context-shared genetic

effects, and the stimulation-specific effects correspond to the context-specific effects. Put simply, we propose the context-shared genetic effects be considered a "baseline" effect, and that the context-shared genetic effects are simply offsets to the context-shared effect. This model is directly related to equation (5):

$$E_{ijt_i} = (E_{ij.}) + (E_{ijt_i} - E_{ij.}) \tag{6}$$

where $E_{ij.}$ and $(E_{ijt_i} - E_{ij.})$ correspond to the context-shared and context-specific genetic effects respectively. By construction, $E_{ij.}$ and $(E_{ijt_i} - E_{ij.})$ are orthogonal, and thus we have generated orthogonal components for the context-shared and context-specific components of expression.

Hierarchical false discovery correction Multiple hypotheses correction in the context of discovering genes, gene-context pairs, and downstream associations of genetically-regulated gene expression with phenotypes varies across approaches [3, 4, 5]. For discovering gene and gene-context associations, previous approaches often leverage a Bonferroni correction when investigating a single context, and may use FDR within a context when investigating multiple contexts [4, 5]. After conducting an association test between a phenotype and genetically regulated gene expression, an additional Bonferroni correction is often employed across all tested expression-context-phenotype trios [5]. As this approach across all expression-context-phenotype trios may be too stringent, FDR may also be used. However, adjusting for the FDR within each context or across all contexts simultaneously may lead to an inflation or deflation to the false discovery proportion within certain contexts [6].

To simultaneously control the FDR across all contexts at once, a hierarchical false discovery correction—treeQTL—was developed [6]. Though treeQTL was originally developed for use in eQTL studies, its properties hold for any false discovery correction where such a hierarchy (e.g. gene level and gene-context level) exists[7]. Briefly, TreeQTL first combines all gene-context p-values for a given gene simultaneously using Simes's procedure (other related procedures may also be used) to determine if there is an association at this given locus. If there is an association at the locus, FDR is then employed across the contexts within that gene. Importantly, if a gene does not have a significant association as determined by the first step, contexts are not included in the additional correction procedure, thus decreasing the number of tests that need to be accounted for in multiple correction. This approach has been shown to properly control the false discovery rate across an arbitrary number of contexts and levels in the hierarchy, making it an invaluable tool in the context of gene, gene-context, and gene-context-trait discoveries.

To properly adjust the FDR for CONTENT, we use a hierarchy of 3 levels; (1) at the level of the gene, (2) at the level of the context, and (3) at the level of the method or model.

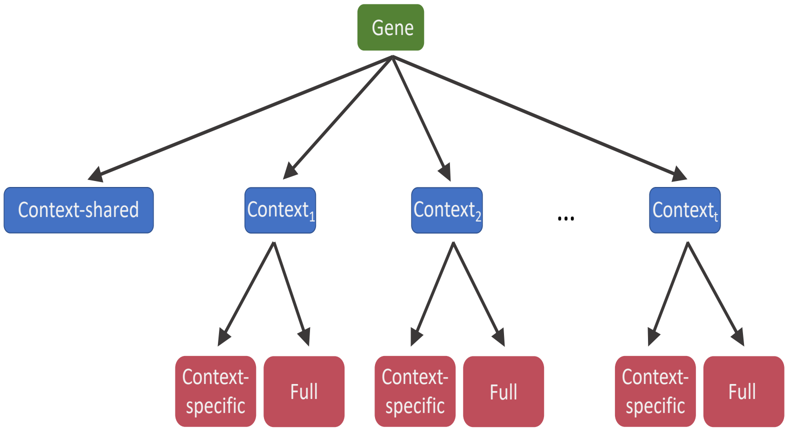

Supplementary Figure 3. Hierarchical false discovery correction. Here, we show the structure of the hypothesis tests for determining whether a gene has a heritable component. A gene (green, top level) is considered heritable if it has a heritable context-shared component or if it was heritable for a specific context (blue, second level). A given gene-context may be heritable due to either the full or context-specific model of CONTENT (red, third level).

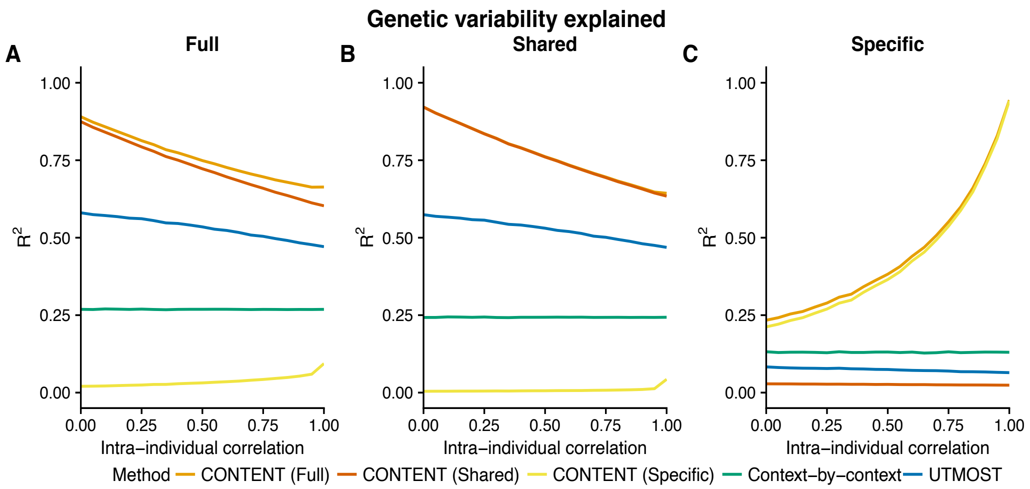

Supplementary Figure 4. CONTENT is powerful and well-calibrated in simulated data. Accuracy of each method to predict the genetically regulated gene expression of each gene-context pair for different correlations of intra-individual noise across contexts. Mean adjusted R^2 across contexts between the true (A) full, (B) shared, and (C) specific genetic components of expression and the predicted component for each method and for different levels of intra individual correlation. We show here the accuracy for each component and method for all gene-contexts pairs, regardless of whether they had only context-shared or had both context-shared and context-specific effects. Notably, 75% of gene-contexts did not have a context-specific effect, and therefore CONTENT(Shared) captures nearly all of the full variability in these contexts (i.e. the full model is comprised of only shared effects). Further, as only 25% of gene-contexts had context-specific effects, CONTENT(Specific) on average captures very little of the full variability.

Supplementary Note 1

Simulations under additional parameter settings In this section, we evaluate CONTENT, UTMOST, and the context-by-context approach using the same simulations framework as in the main text (Figure 2), however here we show each methods' performance while varying additional parameters (Supplementary Figure 5). We also show the performance of each method when the heritability of the context-shared and context-specific effects are equal (.2; Supplementary Figure 6) and where the context-shared heritability is less than the context-specific effects (.1 and .3 respectively; Supplementary Figure 7)).

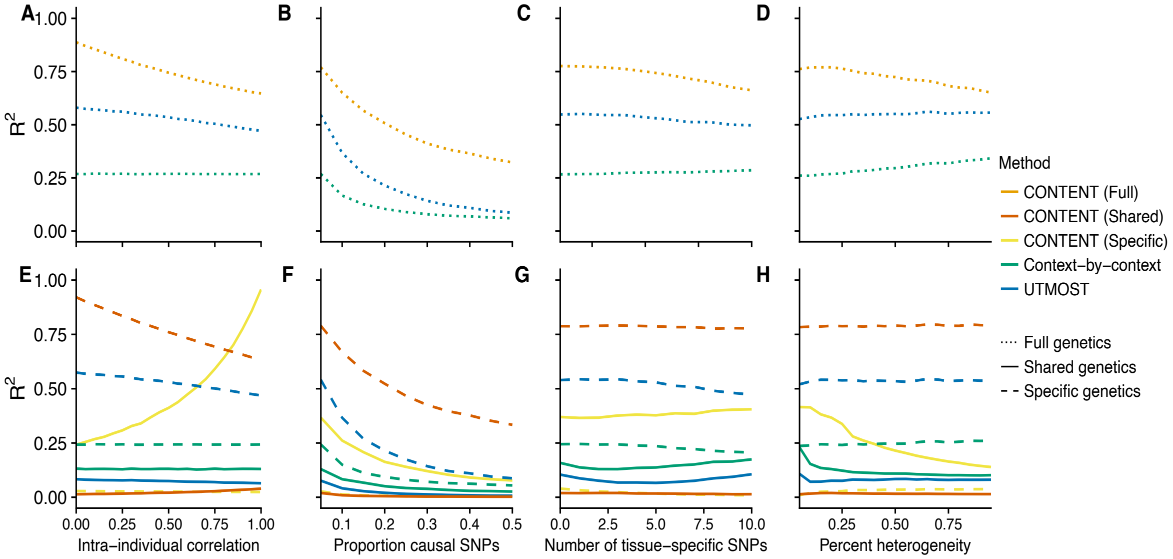

Supplementary Figure 5. Prediction accuracy across simulated data with higher context-shared than context-specific heritability (.3 and .1 respectively). Under a simulations framework, we evaluated the performance of each method to predict the total expression using the mean adjusted R^2 for each gene-context pair across all iterations for different (A,E) correlation between contexts, (B,F) proportion of causal cis-SNPs, (C,G) number of context-specific SNPs, and (D,H) the percent of contexts with context-specific effects on top of the shared effects. (A-D) show the correlation between the true full (specific + shared) genetic component and the estimated full genetic component of each method, and (E-H) show the correlations of the true genetic shared and specific genetic components of the output of each method (where CONTENT separates the two).

For all methods, the baseline of parameters was .3 shared heritability, .1 specific heritability, 500 cis-SNPs, 20 contexts, 0 correlation between contexts, .05 percent causal SNPs, 2 context-specific SNPs, and 20% specificity (signifying the overlap with the shared effects, as well as the percent of contexts with a specific effect). CONTENT continued to outperform the previous methods, and UTMOST consis-

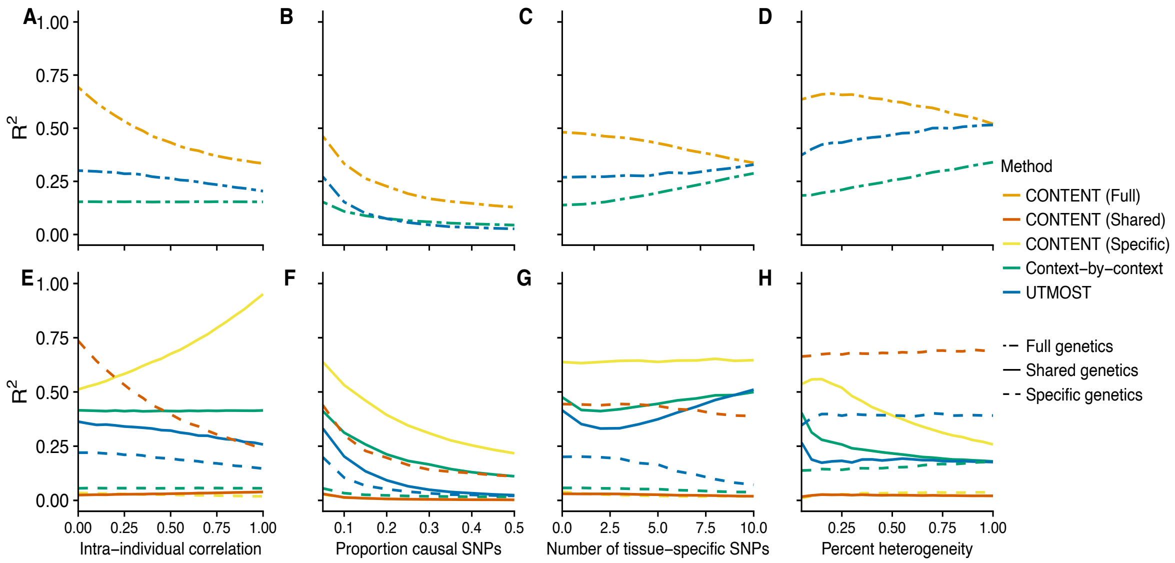

Supplementary Figure 6. Prediction accuracy across simulated data with equal context-shared and context-specific heritability (.2). Under a simulations framework, we evaluated the performance of each method to predict the total expression using the mean adjusted R^2 for each gene-context pair across all iterations for different (A,E) correlation between contexts, (B,F) proportion of causal cis-SNPs, (C,G) number of context-specific SNPs, and (D,H) the percent of contexts with context-specific effects on top of the shared effects. (A-D) show the correlation between the true full (specific + shared) genetic component and the estimated full genetic component of each method, and (E-H) show the correlations of the true genetic shared and specific genetic components of the output of each method (where CONTENT separates the two).

tently outperformed the context-by-context approach. UTMOST consistently performed better than the context-by-context approach, likely as this simulation framework better fits the model's assumptions. We note that UTMOST performed better than CONTENT when there were context-specific effects across all contexts (and this set of effects lied on top of SNPs with a shared effect) and the heritability of context-specific effects dominated the heritability of context-shared effects (Supplementary Figure 7). Given our analysis of GTEx data this architecture may not be entirely common, however this provides further evidence that each method may outperform the other under different architectures, and should therefore be used in complement with the others.

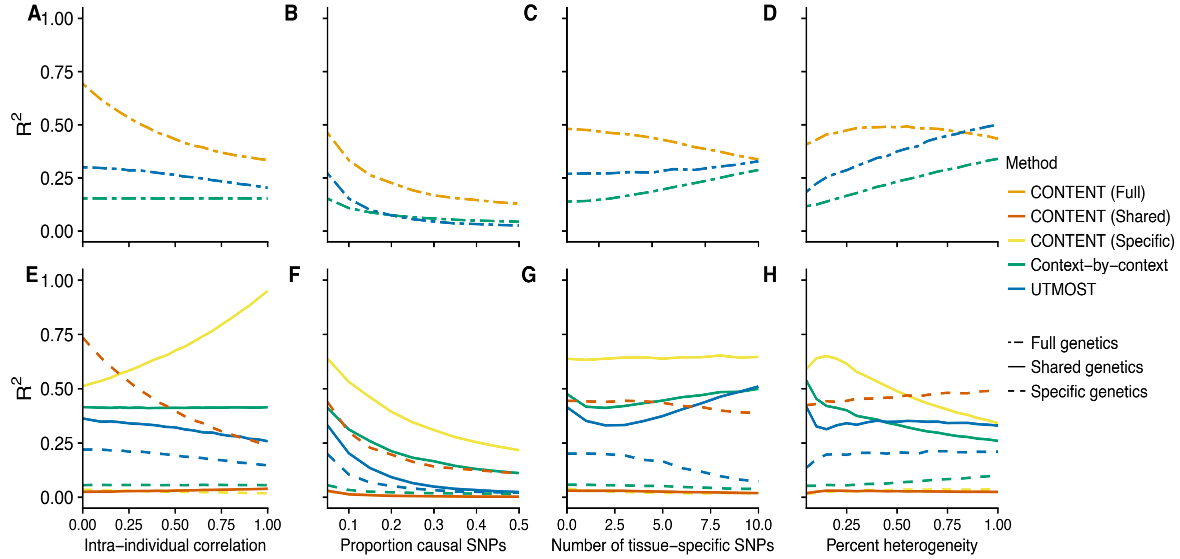

Supplementary Figure 7. Prediction accuracy across simulated data with lower context-shared than context-specific heritability (.1 and .3 respectively). Under a simulations framework, we evaluated the performance of each method to predict the total expression using the mean adjusted R^2 for each gene-context pair across all iterations for different (A,E) correlation between contexts, (B,F) proportion of causal cis-SNPs, (C,G) number of context-specific SNPs, and (D,H) the percent of contexts with context-specific effects on top of the shared effects. (A-D) show the correlation between the true full (specific + shared) genetic component and the estimated full genetic component of each method, and (E-H) show the correlations of the true genetic shared and specific genetic components of the output of each method (where CONTENT separates the two).

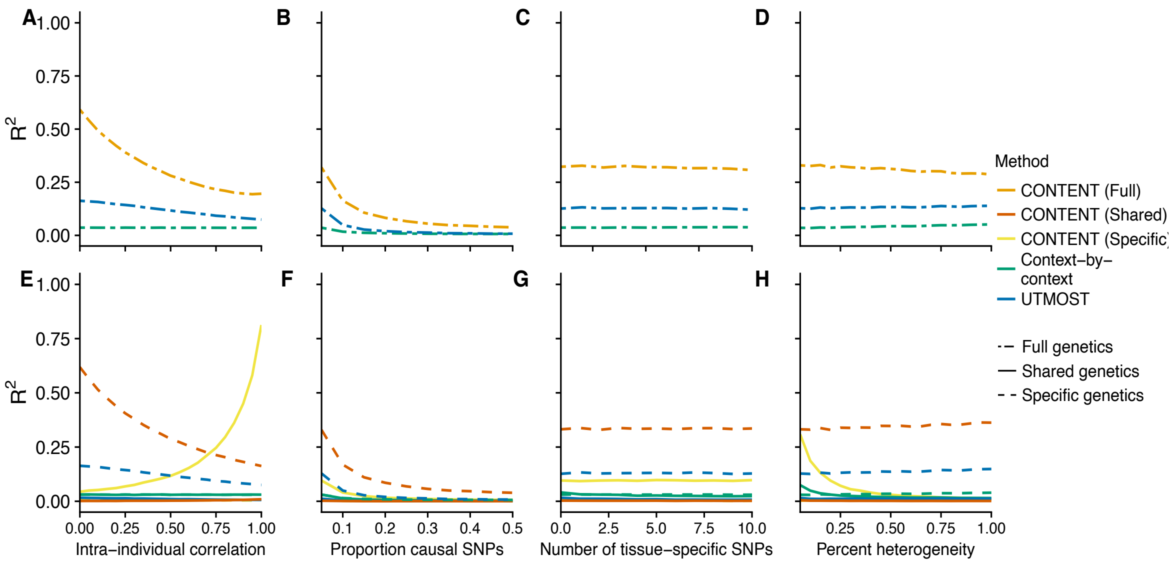

Supplementary Figure 8. Prediction accuracy across simulated data (2,000 cis-SNPs). Under a simulations framework, we evaluated the performance of each method to predict the total expression using the mean adjusted R^2 for each gene-context pair across all iterations for different (A,E) correlation between contexts, (B,F) proportion of causal cis-SNPs, (C,G) number of context-specific SNPs, and (D,H) the percent of contexts with context-specific effects on top of the shared effects. (A-D) show the correlation between the true full (specific + shared) genetic component and the estimated full genetic component of each method, and (E-H) show the correlations of the true genetic shared and specific genetic components of the output of each method (where CONTENT separates the two).

Supplementary Note 2

Runtimes of methods We compared the runtimes and memory requirements of our software that fits both CONTENT and the context-by-context approach (10-fold cross-validation) to UTMOST (5-fold cross-validation). Our software takes advantage of the memory-mapped,fast penalized linear regression framework implemented by R package `bigstatsr` [8]. When we tested both approaches on 100 randomly-selected GTEx genes, not only was the runtime of UTMOST—while running half as many cross-validation folds as our method—on average over 3x the runtime of running our software, but the average memory required by UTMOST was also over 10x the memory required by our software.

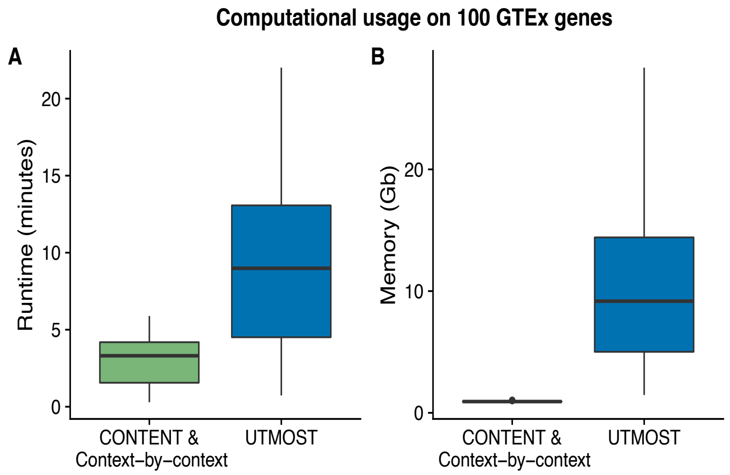

Supplementary Figure 9. Runtime and memory usage of CONTENT and the context-by-context approach compared to UTMOST. We saved the runtime and memory usage (in gigabytes—Gb) for UTMOST and our software that fits both CONTENT and the context-by-context approach on 100 randomly-selected GTEx genes (boxplots represent the minimum, first quartile, median, third quartile and maximum across 100 fit genes). The median runtime and memory usage of running UTMOST was over 3x and 10x the runtime and memory usage of running our software that fits both CONTENT and the context-by-context approach.

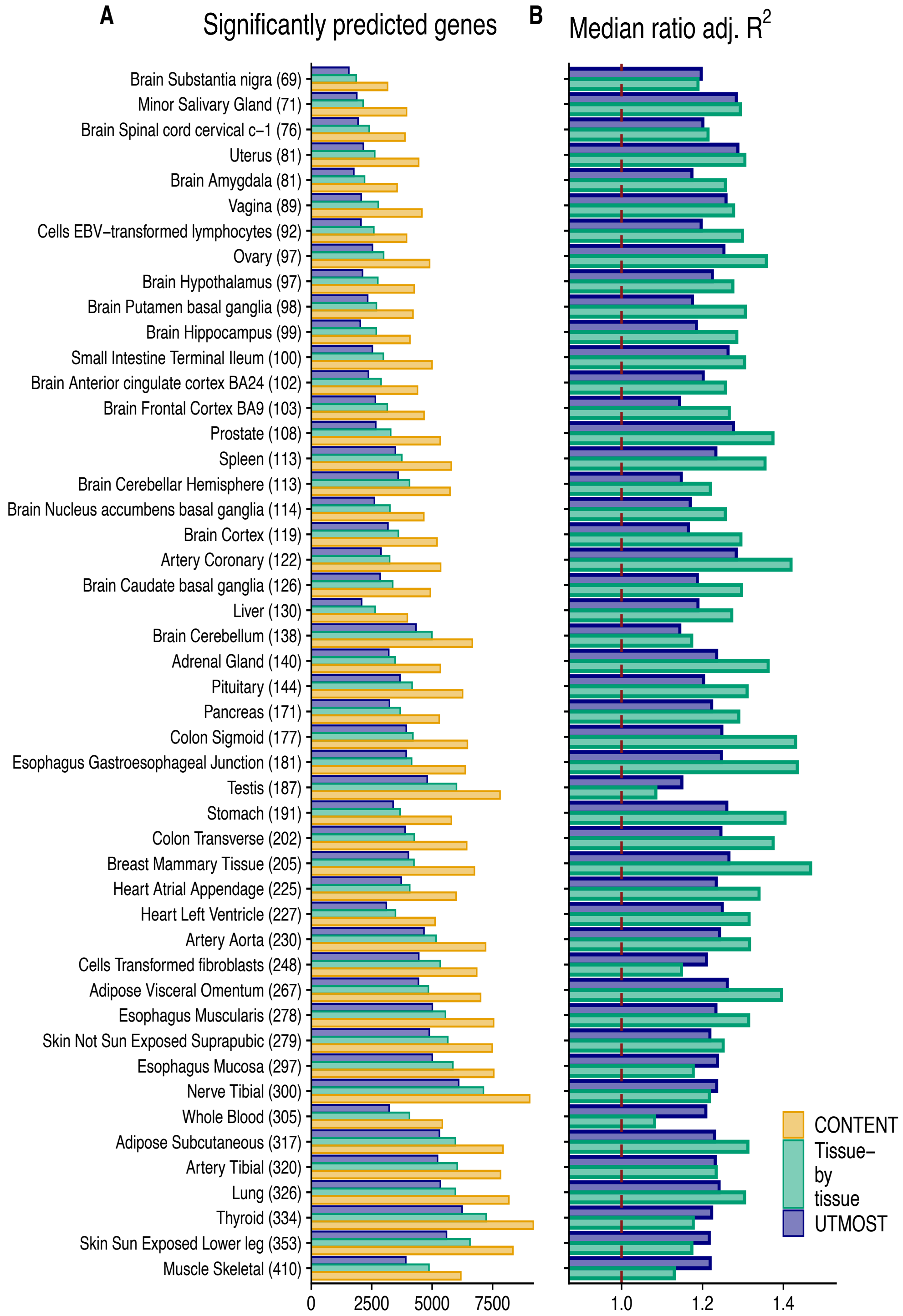

Supplementary Figure 10. Power of CONTENT, UTMOST and the context-by-context model across GTEx on genes run by UTMOST. (A) The number genes of genes with a significantly predictable component across each context with sample size included in parentheses (B) The median ratio of adjusted R^2 (CONTENT/context-by-context,CONTENT/UTMOST) across the union of genes significantly predicted by CONTENT and either the context-by-context model or UTMOST.

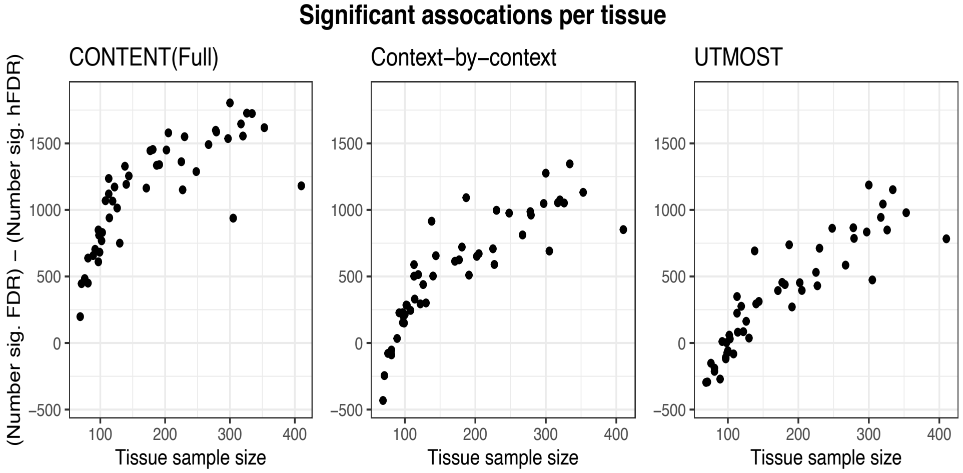

Supplementary Figure 11. The difference in number of eAssociations when using FDR within each context separately and using hFDR across all tissues simultaneously. We show the difference in number of associations when using different FDR strategies as a function of tissue sample size in the GTEx dataset. CONTENT represents the CONTENT(Full) model. The changes are most interpretable using the context-by-context approach, as there is no "borrowing" of power by tissues with a small sample size from tissues with a larger sample size (which may be the case with CONTENT and UTMOST). Interestingly, in tissues with small sample sizes, there is a decrease in the number of significant associations when using FDR rather than hFDR. Conversely, in tissues with larger sample sizes, there is an increase in the number of significant associations.

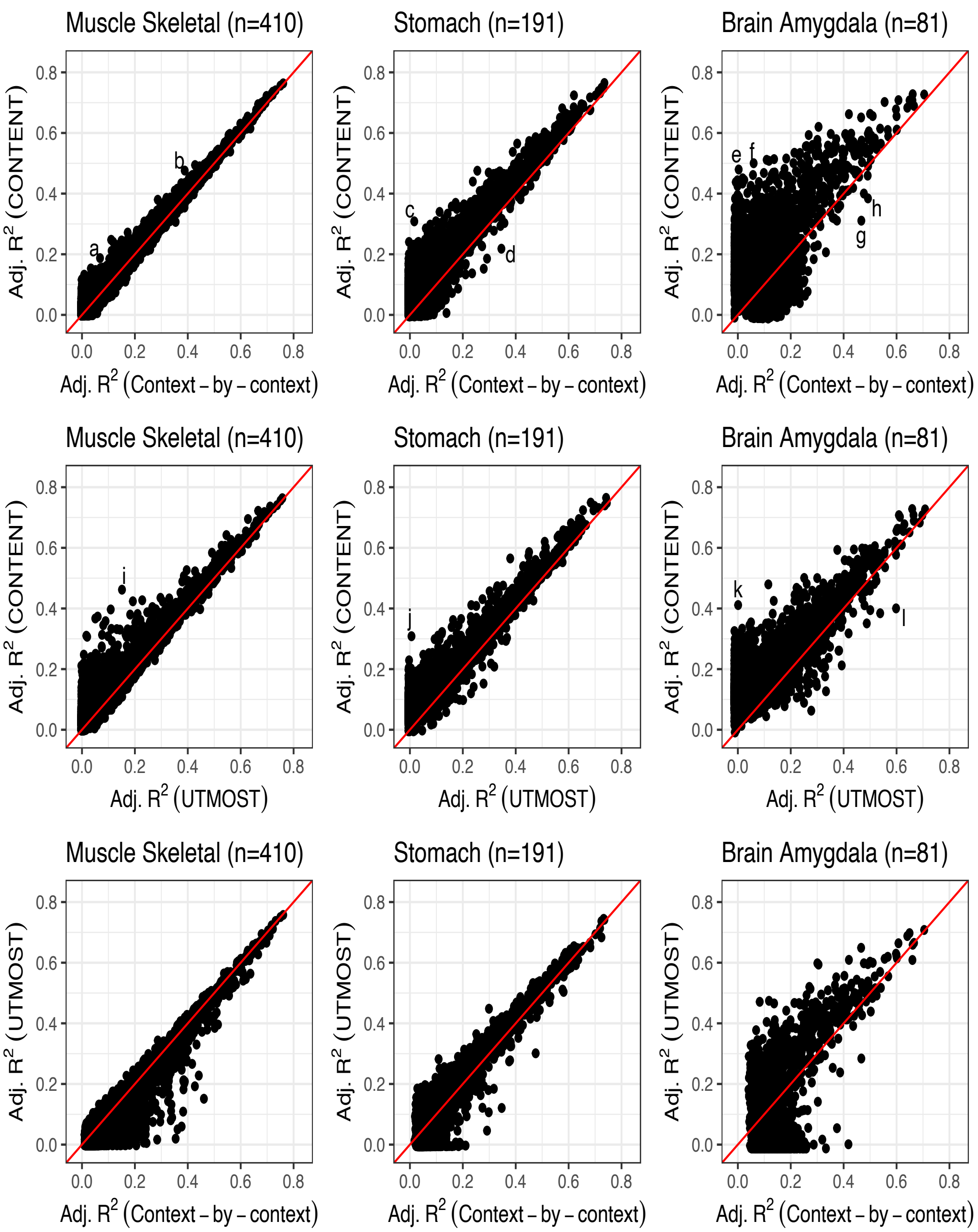

Supplementary Figure 12. Performance of CONTENT, UTMOST, and the context-by-context approach on individual gene-tissue pairs across GTEx tissues. We highlight several genes for which there was a sizable difference in performance between CONTENT and a previous method. (a) ENSG00000188878.12 had a significant CONTENT model in over 46 tissues. The shared component explained an average of 23% observed variability explained whereas the specific component explained an average of 5%. (b) ENSG00000255513.1 was similar to (a), but had a significant model in all 48 tissues and the shared component explained on average 44% of the variability in the observed expression while the specific component explained roughly 1%. (c) ENSG00000160072.15 Also was dominated by the shared variability which explained on average 31% of the variability in the 48 tissues for which a significant content model was built (as opposed to 1% by the specific component).

(d) ENSG00000198203.5 has a large specific component in the stomach explaining 13% of the variability of observed expression. The shared component of expression only explained a significant amount of variability in a subset of tissues in which the gene was expressed, and while it explained a similar amount of variability to the specific component (15%), there was likely heterogeneity in a subset the tissues since several had an insignificant amount of variability explained by the shared component. (e) ENSG00000238142.1 followed a similar trend to (a) and (b), where the shared component dominated the variability explained. (f) ENSG00000119673.10 also followed the same trend as (a), (b), and (c). (g) ENSG00000226314.3 was expressed in over 43 tissues, but only 22 had a significant CONTENT model. The shared component explained on average 7% of the observed variability, and explained much more in fibroblasts, spleen, and tibial nerve. There was a strong specific signal in only brain tissues. There likely was an additional level of heterogeneity that was shared across a subset of tissues rather than all tissues. (h) ENSG00000084628.5 showed a similar trend to (g) in which the shared component explained most of the variability within brain tissues, but was not predictive of expression in non-brain tissues. (i) ENSG00000166454.5 Though this gene was expressed in all 48 tissues, only 6 had significant predictors, and the variability explained in the observed expression was 10% by the specific component and 6% by the shared on average. In skeletal muscle, most of the variability explained was by the specific component. (j) ENSG00000160072.15 Most of the variability explained for the stomach expression came from the specific model (28.5%) than the shared model (1%). (k) ENSG00000153253.11 was expressed in 25 tissues, but only 6 tissues had a significant model. The amygdala in particular had predictors in which all the variability explained came from the CONTENT specific model. (l) ENSG00000261701.2 On average, the shared component explained a large portion of variability in the observed expression of brain tissues. There were large specific components of expression for both brain and non-brain tissues, but there was likely some heterogeneity of shared effects which led to a lower performance of CONTENT compared to UTMOST.

Supplementary Table 1. The number of eAssociations for each tissue and method when employing either FDR within each tissue separately or hFDR across all tissues simultaneously. CONTENT represents the CONTENT(Full) model.

	hFDR			FDR		
**Tissue**	**CONTENT**	**CxC**	**UTMOST**	**CONTENT**	**CxC**	**UTMOST**
Adipose Subcutaneous (317)	7723	6011	5300	9369	7065	6244
Adipose Visceral Omentum (267)	6776	4886	4432	8267	5698	5017
Adrenal Gland (140)	5083	3508	3205	6275	4011	3499
Artery Aorta (230)	7002	5211	4662	8552	6208	5374
Artery Coronary (122)	5054	3261	2885	6226	3555	2970
Artery Tibial (320)	7582	6095	5222	9137	7170	6266
Brain Amygdala (81)	3222	2225	1760	3672	2136	1571
Brain Anterior cingulate cortex BA24 (102)	3984	2917	2364	4752	3202	2423
Brain Caudate basal ganglia (126)	4600	3390	2851	5614	3829	3014
Brain Cerebellar Hemisphere (113)	5348	4110	3592	6468	4699	3941
Brain Cerebellum (138)	6298	5050	4333	7626	5965	5025
Brain Cortex (119)	4897	3628	3172	5963	4142	3448
Brain Frontal Cortex BA9 (103)	4278	3167	2653	5108	3450	2684
Brain Hippocampus (99)	3695	2708	2028	4377	2858	1954
Brain Hypothalamus (97)	3930	2775	2120	4540	2928	2012
Brain Nucleus accumbens basal ganglia (114)	4303	3285	2612	5243	3615	2693
Brain Putamen basal ganglia (98)	3893	2718	2334	4703	2919	2337
Brain Spinal cord cervical (76)	3575	2422	1934	4059	2344	1782
Brain Substantia nigra (69)	2838	1880	1554	3036	1448	1258
Breast Mammary Tissue (205)	6421	4285	4015	8000	4956	4410
Cells EBV-transformed lymphocytes (92)	3638	2612	2057	4342	2839	2068
Cells Transformed fibroblasts (248)	6444	5393	4447	7732	6369	5309
Colon Sigmoid (177)	6220	4238	3930	7665	4863	4385
Colon Transverse (202)	6048	4302	3881	7498	4953	4334
Esophagus Gastroesophageal Junction (181)	6143	4185	3927	7597	4906	4366
Esophagus Mucosa (297)	7191	5917	5006	8727	6965	5840
Esophagus Muscularis (278)	7364	5612	5009	8962	6599	5875
Heart Atrial Appendage (225)	5723	4108	3720	7085	4816	4251
Heart Left Ventricle (227)	4803	3511	3105	5954	4101	3535
Liver (130)	3616	2673	2083	4366	2974	2120
Lung (326)	7851	6023	5340	9578	7075	6189
Minor Salivary Gland (71)	3630	2170	1884	4076	1925	1591
Muscle Skeletal (410)	5773	4908	3910	6954	5760	4693
Nerve Tibial (300)	8791	7201	6097	10595	8477	7284
Ovary (97)	4557	3024	2531	5406	3252	2412
Pancreas (171)	5065	3725	3235	6229	4339	3629
Pituitary (144)	5838	4219	3667	7093	4875	3979

Continued on next page

Supplementary Table 1 continued: The number of eAssociations for each tissue and method when employing either FDR within each tissue separately or hFDR across all tissues simultaneously. CONTENT represents the CONTENT(Full) model.

**Tissue**	hFDR<br>**CONTENT**	 **CxC**	 **UTMOST**	FDR<br>**CONTENT**	 **CxC**	 **UTMOST**
Prostate (108)	5030	3312	2667	6099	3557	2585
Skin Not Sun Exposed Suprapubic (279)	7136	5713	4880	8722	6674	5666
Skin Sun Exposed Lower leg (353)	7972	6633	5594	9590	7765	6573
Small Intestine Terminal Ileum (100)	4594	3013	2526	5420	3227	2468
Spleen (113)	5490	3782	3480	6726	4284	3704
Stomach (191)	5525	3705	3384	6865	4215	3655
Testis (187)	7007	6077	4796	8342	7169	5534
Thyroid (334)	8879	7305	6240	10603	8651	7392
Uterus (81)	4087	2648	2155	4725	2595	1942
Vagina (89)	4185	2792	2065	4842	2826	1794
Whole Blood (305)	4739	4091	3221	5677	4782	3695

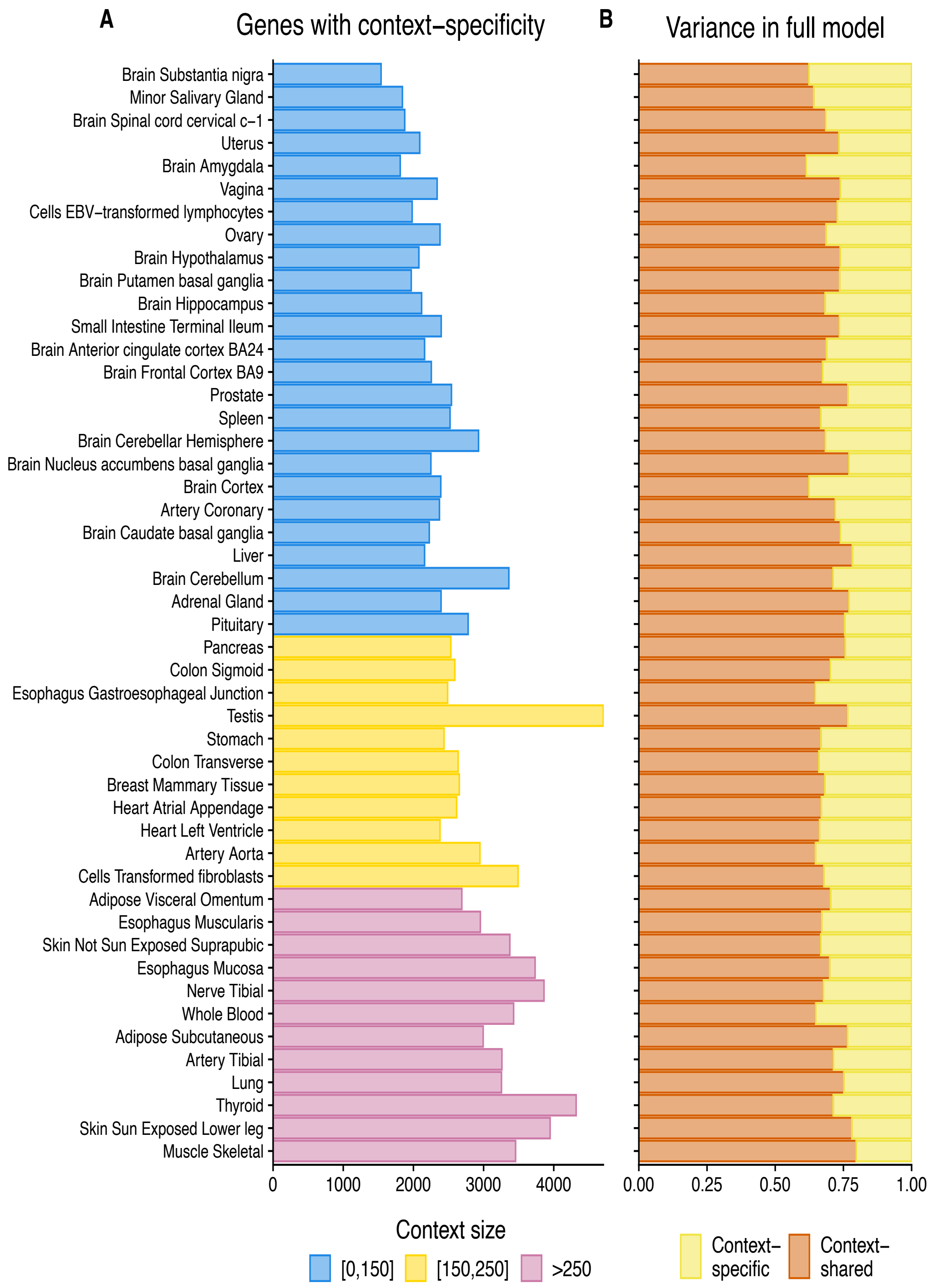

Supplementary Figure 13. PContribution of context-specific genetic regulation in GTEx. (A) The number of genes with a significant (FDR$\leq$ 5%) CONTENT(Specific) model of expression in GTEx. (B) Proportion of expression variance of CONTENT(Full) explained by CONTENT(Specific) and CONTENT(Shared) for genes with a significant CONTENT(Full) model.

Supplementary Note 3

Evaluation TWAS simulations and fine-mapping In this section, we explore the ability of each method to correctly determine the gene-context pair responsible for the association with the phenotype in TWAS. Notably, in these simulations we limited our analyses to situations in which the causal context(s) has been observed. In real data applications, this may not occur, and in such cases, further complexities may arise due to genetic correlation. In these situations, it is likely that all methods will produce false-positive gene-context associations since the true causal context is missing. The complexities posed by missing contexts and cell-types are beyond the scope of this manuscript, and we leave the development of relevant methodology as future work.

Importantly, the models built by CONTENT(Full) can be explained by either the context-shared component, the context-specific component, or both. To implicate a genuine CONTENT(Full) gene-context association (i.e., to elucidate whether a specific context's expression is more strongly associated than the context-shared expression), we propose using only gene-context pairs whose CONTENT(Full) TWAS test statistic is greater in magnitude than the context-shared TWAS test statistic—termed "CONTENT(Fine)." In our simulations we used a test statistics threshold of .5 and found that this heuristic controlled the false positive rate of the CONTENT(Fine) model's associations as well as enriched for correctly-associated contexts.

We evaluated the ability of each method to implicate the correct eAssociation in simulated TWAS data. Across a range of heritability and hetereogeneity (percent of contexts with context-specific genetic effects in addition to the main effects), we simulated 1000 genes for 20 contexts, 100 of which had 3 contexts whose genetic component of expression was associated with the phenotype. We considered sensitivity and specificity as the ability of each method to implicate the correct context for an associated gene. To evaluate sensitivity and specificity, we examined which gene-context pairs were significantly associated with the phenotype after employing the hierarchical false discovery correction [6] as the gene-based false positive rate was well-controlled across methods using this approach.

In the absence of context-shared genetic effects, all methods showed high specificity and sensitivity (Supplementary Figure 15). However, as the genetic variability became more context-shared, the specificity and sensitivity of the context-by-context approach and UTMOST dropped substantially (Supplementary Figure 15). As neither the context-by-context approach nor UTMOST attempt to deconvolve the context-shared and context-specific effect sizes, their weights for a given context contain both context-shared and context-specific signal. Thus when the context-shared effects dominate the heritability, both methods are likely to suggest context-specific associations across all contexts that express an associated gene. The specificity of CONTENT's context-specific component, as well as the full model's weighting of each expression component are paramount to its specificity and sensitivity, as shown by its robust performance across various mixtures of genetic effects (Supplementary Figure 15).

In the GTEx dataset, the fine-mapping TWAS associations produced by our heuristic for the CONTENT(Full) model produced broad associations across many tissues. Though we observed many correct fine-mapping associations for several known gene-trait etiologies (e.g. CYLD and esophagus mucosa in Crohn's [9], LIPC and liver in HDL [10], SORT1 in liver in LDL and HDL [11, 12, 13]), there was not consistent enrichment of a specific tissue known to be relevant for a given trait (for example, the pancreas was not over-represented in associations of Type 2 Diabetes). This could be because the correct tissue or context is missing from the data, horizontal or vertical pleiotropy, or other unknown reasons. As the fine-mapping heuristic performed well in simulated data under a known architecture and where all contexts are observed, we are hopeful that the context-specific estimates will be useful in downstream tissue fine-mapping methods.

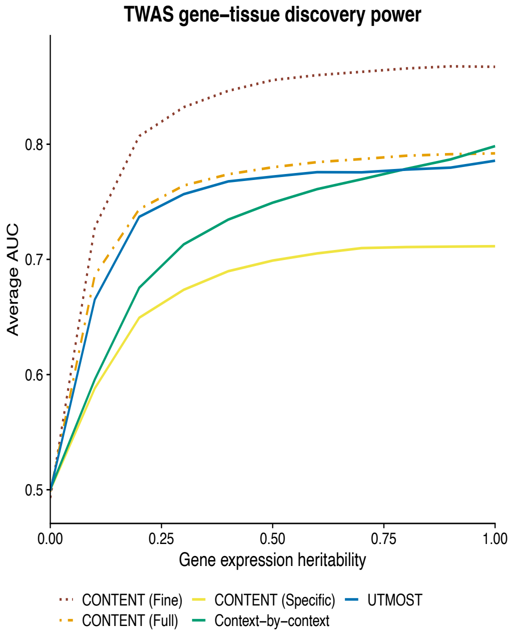

Supplementary Figure 14. Using a heuristic to fine-map CONTENT(Full) associations. Average AUC from 1000 TWAS simulations while varying the overall heritability of gene expression. Each phenotype (1000 per proportion of heritability) was generated from 300 (100 genes and 3 contexts each) randomly selected gene-context pairs' genetically regulated gene expression, and the 300 gene-context pairs' genetically regulated expression accounted for 20% of the variability in the phenotype.

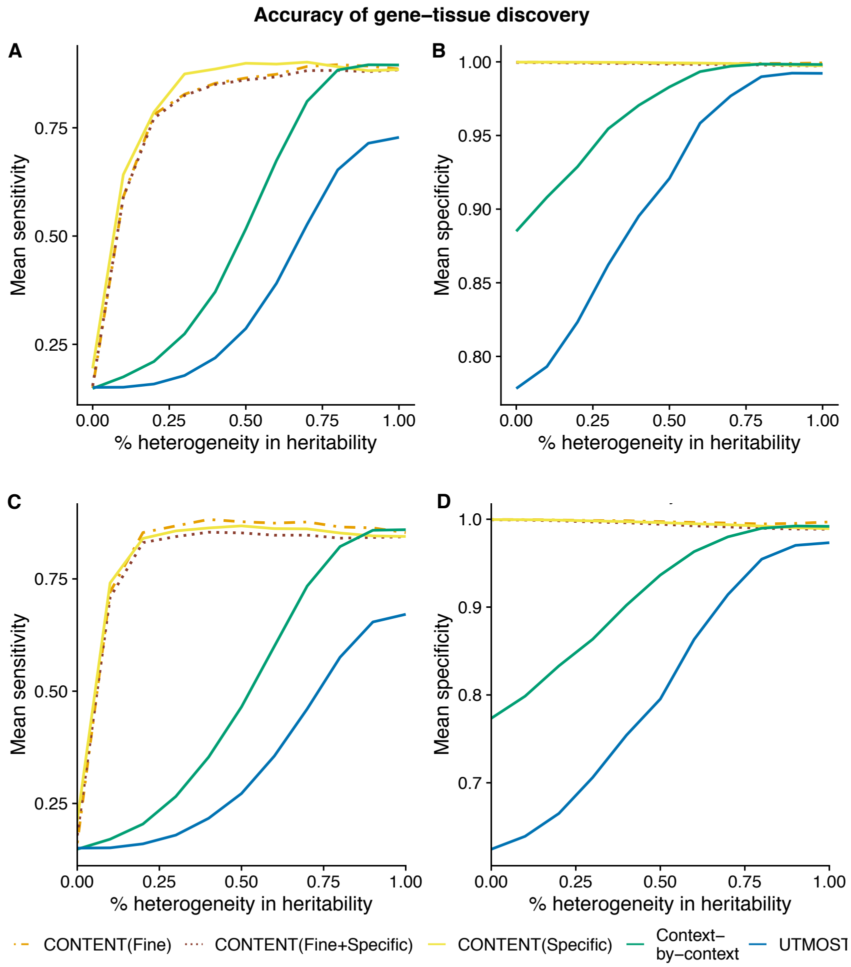

Supplementary Figure 15. CONTENT is sensitive and specific. We simulated 1000 phenotypes from 300 randomly selected gene-tisue pairs' expression while varying the percent heterogeneity and performed a TWAS using the weights output by each method. (A,B) When the total proportion of variability in the phenotype due to the genetically regulated gene expression is .5 and (C,D) when the proportion is .2. The full model of CONTENT was the most sensitive when finding the correct gene-context pair, and is most powerful when there is non-negligible context-specific heritability in addition to the tissue-shared heritability.

Supplementary Table 2. GWAS summary statistics used as input for TWAS. Abbreviation used for each trait as well as its respective study and sample size. The collection of traits from the UKBiobank were self-reported and measured on the same set of individuals across traits.

Symbol	Trait	Study	Sample Size
AD	Alzheimer's disease	Lambert et al. Nat Genet. 2013 [14]	74,046
Asthma	Asthma (self-reported)	UKBB Loh et al. 2018 Nat Genet [15]	361141
Bipolar	Bipolar Disorder	PGC Cell 2018 [16]	73,684
CAD	Coronary Artery Disease	CARDIoGRAM Nat Genet. 2011 [17]	86,995
CKD	Chronic Kidney Disease	Wuttke et al. Nat Genet. 2019 [18]	1,046,070
Crohn's	Crohn's Disease	IIBDGC Europeans Nat Genet. 2015 [19]	13,974
Eczema	Eczema (self-reported)	UKBB Loh et al. 2018 Nat Genet [15]	361,141
FastGlu	Fasting Glucose	MAGIC Nat Genet. 2012 [20]	96,496
HDL	High-density Lipoprotein	Teslovich et al. Nature 2010 [21]	99,900
IBS	Irritible bowel syndrome (self-reported)	UKBB Loh et al. 2018 Nat Genet [15]	361,141
LDL	Low-density lipoprotein	Global lipids genetics consotrium Nat Genet 2013 [22]	188,577
Lupus	Systemic Lupus Erythromous	Bentham et al. Nat Genet 2015 [23]	23,210
MDD	Major Depression Disorder	PGC; Howard et al. Nat Neuro 2019 [24]	807,553
MS	Multiple Sclerosis (self-reported)	UKBB Loh et al. 2018 Nat Genet [15]	361,141
PBC	Primary biliary cirrhosis	Cordell et all. Nat Comm 2015 [25]	13,239
Psoriasis	Psoriasis (self-reported)	UKBB Loh et al. 2018 Nat Genet [15]	361,141
RA	Rheumatoid Arthritis	Okada et al. Nature 2013 [26]	103,638
Sarcoidosis	Sarcoidosis (self-reported)	UKBB Loh et al. 2018 Nat Genet [15]	361,141
Sjogren	Sjogren's Syndrome (self-reported)	UKBB Loh et al. 2018 Nat Genet [15]	361,141
T1D	Type 1 Diabetes	Inshaw et al. Diabetologia 2021 [27]	17,685
T2D	Type 2 Diabetes	DIAGRAM Nat Genet 2018 [28]	898,130
Ulc colitis	Ulcerative Colitis (self-reported)	UKBB Loh et al. 2018 Nat Genet [15]	361,141

Supplementary Table 3. The collection of metabolites and their associated gene(s) (as reported by Ndungu et al. [29]) and TWAS summary statistics.

Metabolite	Gene	Z(CONTENT)	Z(CxC)	Rank(CONTENT)	Rank(CxC)
Alpha-Hydroxyisovalerate	HAO2	NA	NA	Inf	Inf
Arachidonate (20:4n6)	FADS1	19.90	19.50	1.00	1.00
Arachidonate (20:4n6)	FADS2	16.50	14.70	3.00	4.00
Arachidonate (20:4n6)	FADS3	14.60	13.80	4.00	5.00
Asparagine	ASPG	5.18	NA	18.00	Inf
Betaine	BHMT	7.93	7.90	1.00	1.00
Betaine	CBS	6.33	6.04	1.00	1.00
Betaine	CPS1	NA	NA	Inf	Inf
Betaine	SLC6A12	NA	NA	Inf	Inf
Biliverdin	UGT1A1	7.69	NA	5.00	Inf
Bradykinin, des-arg(9)	KLKB1	6.72	5.75	3.00	3.00
Bradykinin, des-arg(9)	KNG1	NA	NA	Inf	Inf
Butyrylcarnitine	ACADS	43.90	48.40	2.00	1.00
Butyrylcarnitine	SLC16A9	6.70	6.92	1.00	1.00
Carnitine	SLC16A9	16.60	17.00	1.00	1.00
Carnitine	SLC22A4	6.66	7.02	3.00	2.00
Carnitine	SLC22A5	5.64	5.42	4.00	6.00
Citrate	SLC13A5	NA	NA	Inf	Inf
Citrulline	ALDH18A1	5.85	6.38	1.00	1.00
Cysteine Glutathione Disulfide	GGT1	7.57	7.87	1.00	1.00
Glutaroyl Carnitine	CPS1	NA	NA	Inf	Inf
Glutaroyl Carnitine	CPT2	9.43	6.40	1.00	2.00
Glutaroyl Carnitine	GCDH	13.90	13.90	1.00	2.00
Glutaroyl Carnitine	SLC7A6	9.35	9.51	2.00	2.00
Glycine	CPS1	7.77	7.88	6.00	6.00
Homocitrulline	SLC7A9	5.27	6.27	3.00	2.00
HWESASXX*	ANPEP	NA	NA	Inf	Inf
Hydroxyisovaleroyl Carnitine	MCCC1	7.97	8.54	2.00	1.00
Indolelactate	CCBL1	NA	NA	Inf	Inf
Inosine	NT5E	7.73	7.78	1.00	1.00
Isobutyrylcarnitine	SLC22A1	NA	5.77	Inf	7.00
Kynurenine	SLC7A5	9.98	6.93	1.00	2.00
Leucine	PPM1K	5.27	NA	3.00	Inf
Myo-Inositol	ISYNA1	NA	NA	Inf	Inf
Myo-Inositol	SLC5A11	6.02	7.07	2.00	1.00
Octadecanedioate	SLCO1B1	NA	NA	Inf	Inf
Pantothenate	SLC5A6	NA	NA	Inf	Inf
Phenyllactate (PLA)	GOT2	6.25	6.83	1.00	1.00
Scyllo-Inositol	SLC5A11	6.89	8.65	2.00	2.00
Serine	CPS1	NA	NA	Inf	Inf
Serine	PHGDH	9.56	10.00	1.00	1.00
Serine	PSPH	NA	NA	Inf	Inf

Continued on next page

Supplementary Table 3 continued: The collection of metabolites and their associated gene(s) (as reported by Ndungu et al. [29]) and TWAS summary statistics.

Metabolite	Gene	Z(CONTENT)	Z(CxC)	Rank(CONTENT)	Rank(CxC)
Succinylcarnitine	CRAT	8.48	8.45	1.00	2.00
Succinylcarnitine	SUCLG2	5.73	NA	1.00	Inf
Tryptophan	SLC16A10	NA	NA	Inf	Inf
Tryptophan	TDO2	NA	NA	Inf	Inf
Tryptophan Betaine	SLC22A4	6.75	6.29	2.00	5.00
Tryptophan Betaine	SLC22A5	6.68	5.94	4.00	7.00
Tyrosine	SLC16A10	NA	NA	Inf	Inf
Urate	SLC2A9	9.98	10.36	2.00	1.00
Uridine	TYMP	7.14	7.52	2.00	2.00
1-Linoleoylglycerol (1-Monolinolein)	APOA5	NA	NA	Inf	Inf
1-Palmitoylglycero-phosphoethanolamine	LIPC	6.01	7.91	3.00	1.00
2-Aminobutyrat	PPM1K	NA	NA	Inf	Inf
2-Aminobutyrat	SLC1A4	8.76	9.44	1.00	1.00
3-Dehydrocarnitine*	SLC22A4	10.60	11.10	2.00	2.00
3-Dehydrocarnitine*	SLC22A5	9.59	8.94	4.00	3.00
5-Oxoproline	OPLAH	19.60	19.80	9.00	8.00

Supplementary Note 4

TWAS discoveries as a function of heritability thresholding. In the main text, we put forth all gene-context pairs that were genetically predicted with a nominal pvalue of .1. As the procedure we use for false discovery adjustment was robust across contexts, we evaluated the number of discoveries that are potentially made when raising the threshold for the nominal pvalue. Our results suggest that there may be minimal correlation between genetic-predictability and strength of TWAS association.

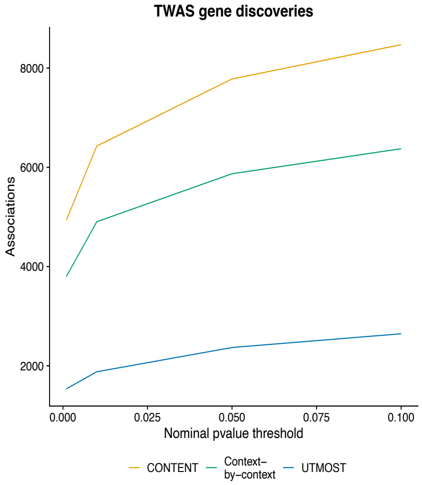

Supplementary Figure 16. TWAS discoveries across predictability thresholds. The number of hierarchical-FDR-corrected TWAS discoveries as a function of the nominal pvalue cutoff for a given gene-tissue's cross-validation expression prediction.

Supplementary Note 5

CONTENT can accommodate additional levels of pleiotropy among contexts While the original model of CONTENT enables a simple decomposition into a component that is shared across all contexts and another that is specific to a single context, there may be cases in which additional sharing exists across a subset of contexts. For example, the group of brain tissues measured in the GTEx consortium have shown similar patterns in terms of cis-genetic variability [30, 31, 32] as well as intra-individual residual correlations (Supplementary Figure 1). To further disentangle the shared and tissue-specific genetic components of expression in the brain tissues, we added an additional term to the CONTENT decomposition which accounts for genetic effects that are only shared across the brain tissues. In more detail, we decompose the original context-shared component of expression into a new context-shared component that is shared across all tissues and a brain-shared component that is shared across only the brain tissues:

$$E_{j\cdot} = E'_{j\cdot} + E_{j\dot{b}} \tag{7}$$

Here, $E'_{j\cdot}$ (the new context-shared term) is an intercept, $E_{j\dot{b}}$ (the brain-shared term) is the effect size on an indicator variable for brain tissues, and estimates of both terms are generated for each individual using a simple linear regression. While introducing an additional term for the shared component will increase the resolution of the model, i.e. the novel model may discover new components of brain-sharing that were miscategorized as tissue-specific in multiple brain tissues, there may be a significant loss in power as this decomposition is only possible for individuals who have been sampled in both multiple brain and non-brain tissues. Additionally, under this decomposition, the full model for brain tissues contains three terms—the context-specific, brain-shared, and globally shared—resulting in a loss of a degree of freedom relative to the original model.

To evaluate the effect of an additional source of effects-sharing on the performance of CONTENT, we simulated an additional genetic effect that lied on top of a subset of SNPs with a main, overall context-sharing effect in 25% of the contexts. As the heritability of this additional source of sharing grew, the context-specific component of CONTENT began to capture variability due to both the context-specific and secondary context-shared effects (Supplementary Figure 17). When we used CONTENT brain, the context-specific component of CONTENT no longer produced predictors that captured variability due to the additional source of effects-sharing (mean R^2 of true brain effects and predicted tissue-specific effects dropped from 0.127 to 0.004 across simulations), and the component responsible for capturing the additional source of effects-sharing–CONTENT(Brain)– was robust (average R^2 between true and predicted brain-shared effects 0.49).

We applied the CONTENT brain model to GTEx, but note that such a component is only identifiable for individuals who have been sampled in both multiple brain and multiple non-brain tissues. For our analysis of the GTEx data, our sample size decreased to 12,904 genes, 26 tissues, and 150 individuals when using CONTENT brain. In general, using this model, the number of genetic tissue-specific components in the brain tissues decreased (Supplementary Figure 18). Of the genes that were implicated in the original CONTENT model as having a tissue-specific component but were no longer captured in the CONTENT brain model with a tissue-specific component, roughly 12% overlapped with the genes implicated by the additional brain-shared component. The CONTENT brain model discovered 4,811 genes with an overall tissue-shared component as well as 1,960 genes with a brain-shared components (of which 66% also had an overall tissue-shared component). The prediction accuracy was similar in both the original and brain models of CONTENT (Supplementary Figure 19).

We next compared the performance of the original CONTENT model to the CONTENT brain model in TWAS using simulated data (generated as aforementioned) as well as GTEx. While the mean AUC between both methods was similar in the simulated data, CONTENT brain was more sensitive than the original CONTENT model when shared brain effects existed (Supplementary Figure 20). Further, despite the fact that the sample size and number of tissues in GTEx data subsetted for the brain model is smaller, CONTENT discovered a non-trivial additional number of TWAS associations (Supplementary Table 4). In several neurological disorders, the number of context-specific genes decreased when using

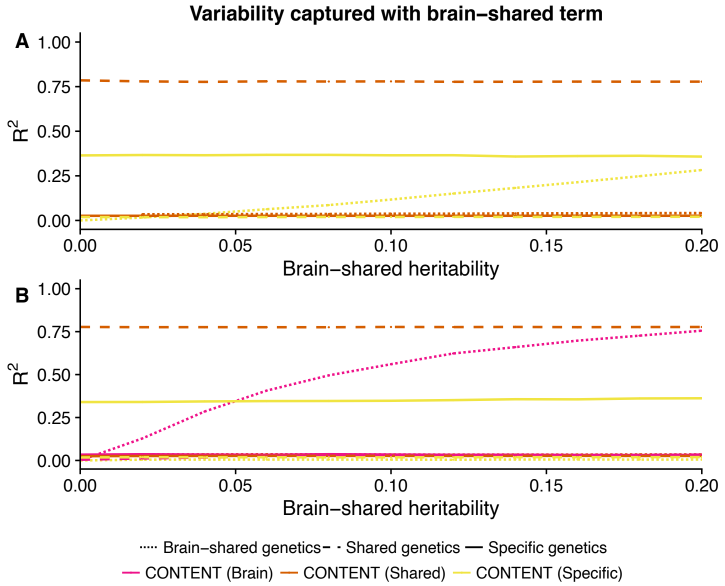

Supplementary Figure 17. Additional sources of tissue-sharing may confound the tissue-specific component. (A) The original CONTENT model without accounting for the additional source of shared genetic effects when such a component exists. (B) When we introduce an additional shared component to the CONTENT model, CONTENT(Brain), the specific component does not capture this additional component, and the additional component is recovered.

the brain model, however the brain model discovered genes whose genetics were shared across only the brain-shared component (Supplementary Table 4). When we examined previous TWAS associations, such as APOC1 and AD, the original CONTENT approach showed association with the thyroid. However, this signal was removed using the brain-pleiotropy approach and the brain pleiotropic component showed significant association (p=2.20e-23). We observed a similar trend with APOE, where the original CONTENT model implicated several brain tissue associations but no significant shared association. The brain pleiotropy model in turn discovered a brain-tissue-shared component with significant evidence of association (p=2.47e-29). Both genes are known to have neuronal roles in Alzheimer's disease [33].

Performance in GTEx when using the brain component We ran the original and brain versions of the CONTENT model on 12904 genes in 26 tissues and 150 individuals in the GTEx dataset. These individuals were measured in at least 3 brain and non-brain tissues. Interestingly, each model discovered eGenes that were not discovered by their counterpart. The amount of variability was roughly the same in both versions of the model, but the adjusted R^2 was slightly higher in non-brain tissues and slightly lower in brain tissues in the brain model. Importantly, the brain tissues in the brain model have 3 explanatory variables and therefore suffer a larger penalty in the adjusted R^2 relative to the original CONTENT model. The adjusted R^2 improved in the non-brain tissues however, suggesting that the context-shared and context-specific components may be less confounded by the brain tissues in the brain model than in the original model.

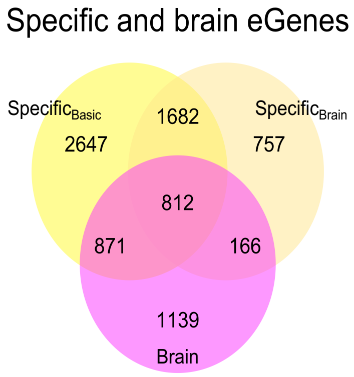

Supplementary Figure 18. Additional sources of effects-sharing may confound the context-specific component. When we run the original CONTENT model and the CONTENT model with the brain-sharing on GTEx genes that are expressed in at least 3 brain and 3 non-brain tissues, many of the previous genetic context-specific components in the brain tissues are absorbed by the additional brain-sharing across brain tissues.

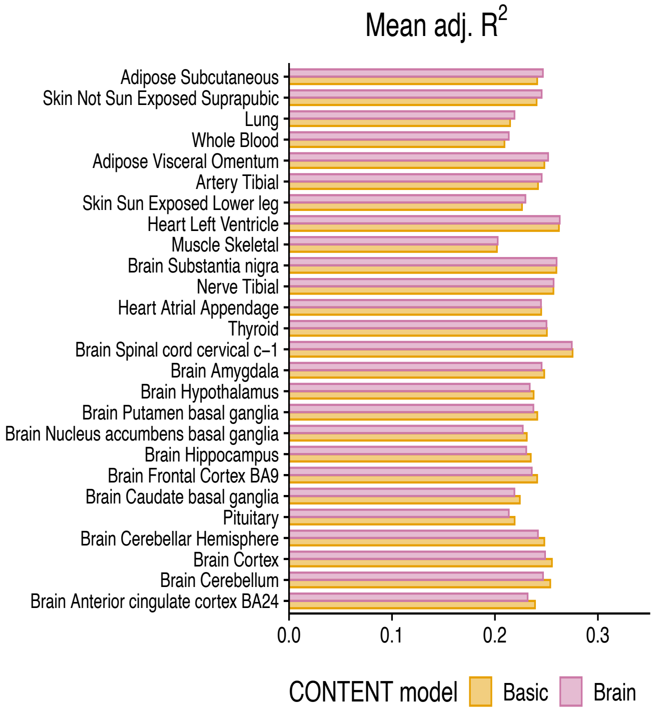

Supplementary Figure 19. Prediction accuracy across tissues in the brain and original CONTENT model. The difference in adjusted R^2 in the brain and original CONTENT(Full) models. While the variability explained is markedly similar in both versions of the model, the adjusted R^2 generally increased in non-brain tissues, and decreased in the brain tissues in the brain model.

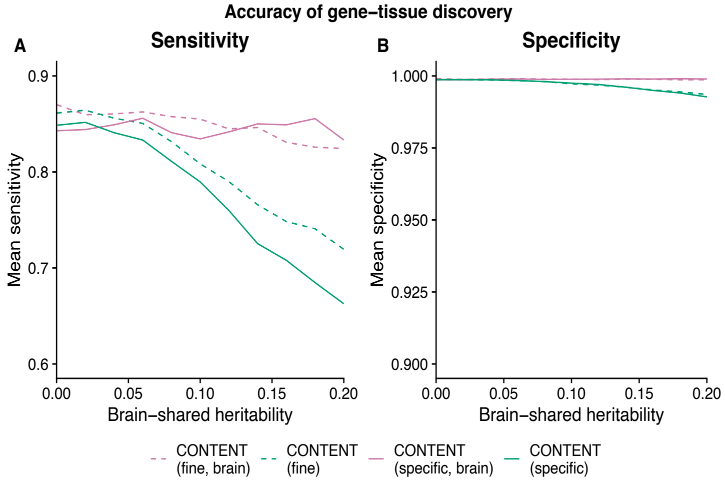

Supplementary Figure 20. Simulated TWAS with brain-shared genetic effects. While the AUC and specificity of the original CONTENT model (green) and the CONTENT model that accounts for brain-shared effects (pink) were nearly the same, the sensitivity was improved when using the brain version of CONTENT in simulated TWAS where there exists brain-shared effects.

TWAS eGenes discovered using the brain version of CONTENT We performed TWAS using weights trained by the original and brain versions of the CONTENT model on 26 tissues, 12,094 genes, and 150 individuals in the GTEx dataset for 17. These individuals were measured in at least 3 brain and non-brain tissues, leading the sample size to be smaller than when using the total GTEx data without any such constraint. While the brain version of the CONTENT model discovered more TWAS eGenes than the original model, the brain model discovered fewer context-specific eGenes than the original model.

Supplementary Table 4. eGenes discovered by each component of CONTENT model in the brain and original models. In total, there were fewer genes discovered using the brain model of CONTENT, however our simulations show that the brain model of CONTENT may improve the resolution of associations. Abbreviations are as follows: AD, Alzheimer's disease; CAD, Coronary Artery Disease; CKD, Chronic Kidney Disease; Crohn's, Crohn's Disease; FastGlu, Fasting Glucose; GFR, Glomerular filtration rate; HDL, High-density lipoprotein; IBS, Irritable bowel syndrome; LDL, Low-density lipoprotein; Lupus, Systemic lupus erythematosus; MDD, Major depressive disorder; MS, Multiple sclerosis; PBC, Primary biliary cholangitis; RA, Rheumatoid arthritis; Sjogren, Sjögren's syndrome; T1D, Type 1 diabetes; T2D Type 2 diabetes; TG, Triglycerides; Ulc colitis, Ulcerative colitis.

	CONTENT original				CONTENT brain				
Trait	CONTENT (All)	CONTENT (Full)	CONTENT (Specific)	CONTENT (Shared)	CONTENT (All)	CONTENT (Full)	CONTENT (Specific)	CONTENT (Shared)	CONTENT (Brain)
AD	76	62	64	19	67	51	59	10	8
Asthma	594	415	487	74	545	386	412	81	39
Bipolar	75	49	47	18	78	43	47	14	8
CAD	13	11	7	2	14	9	11	2	1
CKD	58	39	47	14	51	34	29	15	2
Crohn's	279	205	231	48	265	177	190	46	20
Eczema	109	66	84	4	78	53	61	7	5
FastGlu	65	44	58	5	65	45	45	10	8
GFR	1721	1243	1428	357	1550	1087	1167	313	168
HDL	247	175	217	37	228	116	170	45	19
IBS	14	10	5	2	12	9	3	1	0
LDL	506	380	437	77	477	331	391	74	45
Lupus	356	268	309	73	315	249	245	59	42
MDD	250	155	182	44	189	121	109	43	18
MS	114	94	98	19	114	91	100	21	6
PBC	204	147	170	32	194	137	147	36	23
Psoriasis	180	158	163	39	183	153	152	39	23
RA	286	230	251	85	274	212	231	82	44
Sarcoidosis	90	69	75	10	90	57	73	6	7
Sjogren	24	13	18	2	19	8	14	1	1
T1D	359	303	323	92	311	255	272	101	59
T2D	514	352	422	91	451	310	327	94	32
TG	3251	2429	2791	641	3079	2169	2452	624	299
Ulc colitis	35	28	27	3	16	12	10	2	0

Supplementary References

[1] Yang, J., Lee, S., Goddard, M. & Visscher, P. GCTA: A Tool for Genome-wide Complex Trait Analysis. *The American Journal Of Human Genetics.* **88**, 76-82 (2011)

[2] Lee, S., Yang, J., Goddard, M., Visscher, P. & Wray, N. Estimation of pleiotropy between complex diseases using single-nucleotide polymorphism-derived genomic relationships and restricted maximum likelihood.. *Bioinformatics.* **28**, 2540-2542 (2012,10)

[3] Gusev, A., et al. Integrative approaches for large-scale transcriptome-wide association studies. *Nature Genetics.* **48** pp. 245 EP - (2016,2)

[4] Gamazon, E., et al. A gene-based association method for mapping traits using reference transcriptome data. *Nature Genetics.* **47** pp. 1091 EP - (2015,8)

[5] Barbeira, A., et al. Exploring the phenotypic consequences of tissue specific gene expression variation inferred from GWAS summary statistics. *Nature Communications.* **9**, 1825 (2018)

[6] Peterson, C., Bogomolov, M., Benjamini, Y. & Sabatti, C. TreeQTL: hierarchical error control for eQTL findings.. *Bioinformatics.* **32**, 2556-2558 (2016,8)

[7] Peterson, C., Bogomolov, M., Benjamini, Y. & Sabatti, C. Many Phenotypes Without Many False Discoveries: Error Controlling Strategies for Multitrait Association Studies.. *Genet Epidemiol.* **40**, 45-56 (2016,1)

[8] Prive, F., Aschard, H., Ziyatdinov, A. & Blum, M. Efficient analysis of large-scale genome-wide data with two R packages: bigstatsr and bigsnpr.. *Bioinformatics.* **34**, 2781-2787 (2018,8)

[9] Mukherjee, S., et al. Deubiquitination of NLRP6 inflammasome by Cyld critically regulates intestinal inflammation. *Nature Immunology.* **21**, 626-635 (2020)

[10] Guerra, R., Wang, J., Grundy, S. & Cohen, J. A hepatic lipase (LIPC) allele associated with high plasma concentrations of high density lipoprotein cholesterol.. *Proc Natl Acad Sci U S A.* **94**, 4532-4537 (1997,4)

[11] Strong, A., Patel, K. & Rader, D. Sortilin and lipoprotein metabolism: making sense out of complexity. *Current Opinion In Lipidology.* **25**, 350-357 (2014,10)

[12] Musunuru, K., et al. From noncoding variant to phenotype via SORT1 at the 1p13 cholesterol locus.. *Nature.* **466**, 714-719 (2010,8)

[13] Goettsch, C., Kjolby, M. & Aikawa, E. Sortilin and Its Multiple Roles in Cardiovascular and Metabolic Diseases.. *Arterioscler Thromb Vasc Biol.* **38**, 19-25 (2018,1)

[14] Lambert, J., et al. Meta-analysis of 74,046 individuals identifies 11 new susceptibility loci for Alzheimer's disease. *Nature Genetics.* **45**, 1452-1458 (2013)

[15] Loh, P., Kichaev, G., Gazal, S., Schoech, A. & Price, A. Mixed-model association for biobank-scale datasets. *Nature Genetics.* **50**, 906-908 (2018)

[16] Ruderfer, D., et al. Genomic Dissection of Bipolar Disorder and Schizophrenia, Including 28 Subphenotypes. *Cell.* **173**, 1705-1715.e16 (2018)

[17] Schunkert, H., et al. Large-scale association analysis identifies 13 new susceptibility loci for coronary artery disease. *Nature Genetics.* **43**, 333-338 (2011)

[18] Wuttke, M., et al. A catalog of genetic loci associated with kidney function from analyses of a million individuals. *Nature Genetics.* **51**, 957-972 (2019)

[19] Liu, J., et al. Association analyses identify 38 susceptibility loci for inflammatory bowel disease and highlight shared genetic risk across populations.. *Nat Genet.* **47**, 979-986 (2015,9)

[20] Manning, A., et al. A genome-wide approach accounting for body mass index identifies genetic variants influencing fasting glycemic traits and insulin resistance. *Nature Genetics.* **44**, 659-669 (2012)

[21] Teslovich, T., et al. Biological, clinical and population relevance of 95 loci for blood lipids. *Nature.* **466**, 707-713 (2010)

[22] Willer, C., et al. Discovery and refinement of loci associated with lipid levels. *Nature Genetics.* **45**, 1274-1283 (2013)

[23] Bentham, J., et al. Genetic association analyses implicate aberrant regulation of innate and adaptive immunity genes in the pathogenesis of systemic lupus erythematosus. *Nature Genetics.* **47**, 1457-1464 (2015)

[24] Howard, D., et al. & Major Depressive Disorder Working Group of the Psychiatric Genomics Consortium Genome-wide meta-analysis of depression identifies 102 independent variants and highlights the importance of the prefrontal brain regions. *Nature Neuroscience.* **22**, 343-352 (2019,3,1), https://doi.org/10.1038/s41593-018-0326-7

[25] Cordell, H., et al. International genome-wide meta-analysis identifies new primary biliary cirrhosis risk loci and targetable pathogenic pathways. *Nature Communications.* **6**, 8019 (2015)

[26] Okada, Y., et al. Genetics of rheumatoid arthritis contributes to biology and drug discovery. *Nature.* **506**, 376-381 (2014)

[27] Inshaw, J., et al. Analysis of overlapping genetic association in type 1 and type 2 diabetes. *Diabetologia.* **64**, 1342-1347 (2021)

[28] Mahajan, A., et al. Fine-mapping type 2 diabetes loci to single-variant resolution using high-density imputation and islet-specific epigenome maps. *Nature Genetics.* **50**, 1505-1513 (2018)

[29] Ndungu, A., Payne, A., Torres, J., Van de Bunt, M. & McCarthy, M. A Multi-tissue Transcriptome Analysis of Human Metabolites Guides Interpretability of Associations Based on Multi-SNP Models for Gene Expression. *The American Journal Of Human Genetics.* **106**, 188-201 (2020)

[30] He, Y., et al. sn-spMF: matrix factorization informs tissue-specific genetic regulation of gene expression. *Genome Biology.* **21**, 235 (2020)

[31] Urbut, S., Wang, G., Carbonetto, P. & Stephens, M. Flexible statistical methods for estimating and testing effects in genomic studies with multiple conditions. *Nature Genetics.* **51**, 187-195 (2019)

[32] GTEx Consortium, et al. Genetic effects on gene expression across human tissues. *Nature.* **550** pp. 204 EP - (2017,10)

[33] Zhou, X., et al. Non-coding variability at the APOE locus contributes to the Alzheimer's risk. *Nature Communications.* **10**, 3310 (2019)
