# Supplementary material for: Multi-context genetic modeling of transcriptional regulation resolves novel disease loci
Source: Nat Commun. 2022 Sep 28;13:5704. doi: 10.1038/s41467-022-33212-0 (PMC9519579; doi:10.1038/s41467-022-33212-0)
Supplement: Supplementary file 3 — Reporting Summary [file 41467_2022_33212_MOESM3_ESM.pdf]

nature portfolio

Corresponding author(s): Mike Thompson, Noah Zaitlen

Last updated by author(s): Jul 20, 2022

Reporting Summary

Nature Portfolio wishes to improve the reproducibility of the work that we publish. This form provides structure for consistency and transparency in reporting. For further information on Nature Portfolio policies, see our Editorial Policies and the Editorial Policy Checklist.

Statistics

For all statistical analyses, confirm that the following items are present in the figure legend, table legend, main text, or Methods section.

n/a	Confirmed	
☐	☒	The exact sample size (*n*) for each experimental group/condition, given as a discrete number and unit of measurement
☐	☒	A statement on whether measurements were taken from distinct samples or whether the same sample was measured repeatedly
☐	☒	The statistical test(s) used AND whether they are one- or two-sided<br>*Only common tests should be described solely by name; describe more complex techniques in the Methods section.*
☐	☒	A description of all covariates tested
☐	☒	A description of any assumptions or corrections, such as tests of normality and adjustment for multiple comparisons
☐	☒	A full description of the statistical parameters including central tendency (e.g. means) or other basic estimates (e.g. regression coefficient) AND variation (e.g. standard deviation) or associated estimates of uncertainty (e.g. confidence intervals)
☐	☒	For null hypothesis testing, the test statistic (e.g. *F*, *t*, *r*) with confidence intervals, effect sizes, degrees of freedom and *P* value noted<br>*Give P values as exact values whenever suitable.*
☒	☐	For Bayesian analysis, information on the choice of priors and Markov chain Monte Carlo settings
☐	☒	For hierarchical and complex designs, identification of the appropriate level for tests and full reporting of outcomes
☐	☒	Estimates of effect sizes (e.g. Cohen's *d*, Pearson's *r*), indicating how they were calculated

Our web collection on statistics for biologists contains articles on many of the points above.

Software and code

Policy information about availability of computer code

Data collection	No software was used for data collection
Data analysis	Data were analyzed using R (3.6.0), GCTA (1.93.1), CONTENT 057a732 May 17 2022 (https://github.com/cozygene/CONTENT), UTMOST 85adf3a June 3 2020 (https://github.com/yiminghu/CTIMP), bigstatsr v1.5.6 (https://github.com/privefl/bigstatsr), FUSION-TWAS 2016 (http://gusevlab.org/projects/fusion/), TreeQTL v2.0 (http://bioinformatics.org/treeqtl/)

For manuscripts utilizing custom algorithms or software that are central to the research but not yet described in published literature, software must be made available to editors and reviewers. We strongly encourage code deposition in a community repository (e.g. GitHub). See the Nature Portfolio guidelines for submitting code & software for further information.

Data

Policy information about availability of data

All manuscripts must include a data availability statement. This statement should provide the following information, where applicable:

- Accession codes, unique identifiers, or web links for publicly available datasets
- A description of any restrictions on data availability
- For clinical datasets or third party data, please ensure that the statement adheres to our policy

GTEx v7 is a publicly available dataset through the GTEx portal (genotypes must be accessed through dbGap permissions, and RNA sequencing is available on the GTEx website). The CLUES dataset is also publicly available at Gene Expression Omnibus accession number GSE174188 and dbGap accession number phs002812.v1.p1. Trained weights for the GTEx v7 dataset and our in-house single-cell RNAseq are available at the TWAS/FUSION repository http://gusevlab.org/projects/fusion/. We provide TWAS summary statistics for all three methods on both datasets (as well as an indicator of whether the association was hierarchical

FDR-adjusted significant) at Zenodo link doi.org/10.5281/zenodo.5209239. We include summary statistics for the associations within GTEx and CLUES at the above Zenodo link.

Field-specific reporting

Please select the one below that is the best fit for your research. If you are not sure, read the appropriate sections before making your selection.

☒ Life sciences ☐ Behavioural & social sciences ☐ Ecological, evolutionary & environmental sciences

For a reference copy of the document with all sections, see nature.com/documents/nr-reporting-summary-flat.pdf

Life sciences study design

All studies must disclose on these points even when the disclosure is negative.

Sample size	We used the largest publical available datasets that our lab had access to. This included the GTEx dataset for bulk RNAsequencing and the CLUES dataset for single-cell sequencing. No power calculations were performed, but we note that GTEx is the largest multi-tissue bulk RNA sequencing dataset and CLUES is the most comprehensive single-cell RNA sequencing dataset. In GTEx, the sample size varied per context but included 519 individuals. For CLUES, all individuals were measured in every context and included 90 individuals.
Data exclusions	We limited our analyses to individuals of European ancestry and preprocessed the data (i.e quality control) is included in the methods section as well as the cited manuscripts. This was to limit the effects of population structure on our findings (a known confounder of multi-ethnic studies).
Replication	We evaluate our findings by considering past publications and show that our findings are consistent with previous literature. For example, we compare our findings to previous reports of heterogeneity using other, cited methodological reports. We replicate our findings of TWAS eGenes in several paragraphs by pulling examples from existing, cited scientific literature. We also replicate our TWAS eGene findings from GTEx in the CLUES datasets for several genes.
Randomization	Randomization was not necessary for our analyses as we were simply performing studies of association across entire cohorts.
Blinding	Blinding was not necessary for our analyses as we were simply performing studies of association across entire cohorts.

Reporting for specific materials, systems and methods

We require information from authors about some types of materials, experimental systems and methods used in many studies. Here, indicate whether each material, system or method listed is relevant to your study. If you are not sure if a list item applies to your research, read the appropriate section before selecting a response.

Materials & experimental systems

n/a	Involved in the study	
☒	☐	Antibodies
☒	☐	Eukaryotic cell lines
☒	☐	Palaeontology and archaeology
☒	☐	Animals and other organisms
☐	☒	Human research participants
☒	☐	Clinical data
☒	☐	Dual use research of concern

Methods

n/a	Involved in the study	
☒	☐	ChIP-seq
☒	☐	Flow cytometry
☒	☐	MRI-based neuroimaging

Human research participants

Policy information about studies involving human research participants

Population characteristics	GTEx data contained individuals with genotypes and gene expression measured across 48 tissues. Further information regarding samples can be found in cited manuscripts. CLUES (the single-cell RNA dataset) was comprised of individuals residing in San Francisco county, California from 2007-2009 that were also diagnosed with systemic lupus erythematosus (SLE). Further details and preprocessing can be found in cited manuscripts.
Recruitment	N/A, we did not recruit study participants.
Ethics oversight	Participants' data is de-identified and did not qualify as Human Subjects Research.

Note that full information on the approval of the study protocol must also be provided in the manuscript.
